# Removal of Subinternal Limiting Membrane Hemorrhage Secondary to Valsalva Retinopathy Using a Fovea-Sparing Internal Limiting Membrane Fissure Creation Technique

**DOI:** 10.1155/2024/2774155

**Published:** 2024-08-13

**Authors:** Yasuyuki Sotani, Hisanori Imai, Maya Kishi, Hiroko Yamada, Wataru Matsumiya, Akiko Miki, Sentaro Kusuhara, Makoto Nakamura

**Affiliations:** ^1^ Department of Surgery Division of Ophthalmology Kobe University Graduate School of Medicine, Kobe, Japan; ^2^ Department of Ophthalmology Kansai Medical University, Hirakata, Japan

**Keywords:** internal limiting membrane peeling, macular hole, subinternal limiting membrane hemorrhage, Valsalva retinopathy, vitrectomy

## Abstract

**Introduction:** Valsalva retinopathy can cause submacular hemorrhage (SMH), which may lead to visual disturbances. SMH can extend into the subinternal limiting membrane (ILM) and vitreous spaces, sometimes occurring concomitantly with full-thickness macular holes (FTMHs). Herein, we describe a case in which sub-ILM hemorrhage was removed without peeling the ILM of the central fovea, thus preserving the foveal ILM.

**Case Presentation:** A 48-year-old female patient developed rapid-onset bilateral visual impairment due to SMH secondary to Valsalva retinopathy. The SMH predominantly consisted of sub-ILM hemorrhage. However, detailed observation was challenging due to the dense sub-ILM hemorrhage in the left eye. Initial best-corrected visual acuity (BCVA) in the right and left eyes were 1.2 and 0.03, respectively. Intravitreal tissue plasminogen activator (tPA) and sulfur hexafluoride (SF6) gas injections were initially administered to displace the SMH in the left eye; however, the SMH could not be successfully displaced. A vitrectomy was then performed. Intraoperatively, an ILM fissure beyond the foveal region was created using ILM forceps. The balanced salt solution was sprayed onto the ILM, and the sub-ILM hemorrhage was drained into the vitreous cavity from the ILM fissure. The surgery successfully displaced the sub-ILM hemorrhage while preserving the foveal ILM. No postoperative complications were observed. Visual acuity remained at 1.2 in the right eye and improved to 1.2 in the left eye 6 months postoperatively.

**Conclusion:** Removing foveal sub-ILM hemorrhage without peeling the foveal ILM can be a viable treatment option to preserve the foveal ILM.

## 1. Introduction

Submacular hemorrhage (SMH) is an ophthalmic emergency that leads to rapid visual impairment [1]. Age-related macular degeneration, retinal artery macroaneurysms (RAMs), high myopia, trauma, proliferative diabetic retinopathy, Terson syndrome, and Valsalva retinopathy can lead to SMH [2]. Valsalva retinopathy is a rare condition characterized by sudden visual decline caused by retinal or preretinal hemorrhage following the rupture of the superficial retinal capillaries [3]. Intense aerobic exercise, heavy lifting, vomiting, twisting, and coughing can rapidly increase intraocular venous pressure due to elevated thoracic or abdominal pressure. Thus, Valsalva retinopathy can occur even in healthy individuals [3–7].

Preretinal hemorrhage can occur in the subinternal limiting membrane (ILM) space, subhyaloid space, or both. Preretinal hemorrhage caused by Valsalva retinopathy resolves spontaneously in many cases [4]; however, even small preretinal hemorrhages, measuring one-disc diameter, may require several months to resolve [8]. The following treatment options can be considered: observation, based on the condition of the patient; combined treatment involving intravitreal injection of expansile gas or tissue plasminogen activator (tPA); neodymium:yttrium–aluminum–garnet laser membranotomy; and pars plana vitrectomy (PPV) with ILM peeling [8–11].

In recent years, due to significant improvements in the safety of PPV, treatment using PPV combined with ILM peeling is preferred, particularly for dense sub-ILM hemorrhage. However, when dense sub-ILM hemorrhage is present, it is challenging to accurately determine the presence of intraretinal and subretinal hemorrhage, as well as the potential existence of full-thickness macular holes (FTMHs) concealed beneath the SMH preoperatively. It is not uncommon for the presence of FTMH to be discovered during the procedure when performing ILM peeling and aspirating the SMH. Although FTMH can be closed in most cases, complete restoration of visual acuity is often not achieved. Thus, the treatment of FTMH secondary to Valsalva retinopathy and its prevention warrant further discussion.

We recently reported a novel PPV technique for sub-ILM hemorrhage secondary to SMH associated with RAM rupture that can preserve the foveal ILM by removing the sub-ILM hemorrhage without peeling the central foveal ILM, and we discussed the possibility of preventing perioperative FTMH formation using this surgical technique [12]. In this report, we describe a case of sub-ILM hemorrhage secondary to Valsalva retinopathy treated using the abovementioned surgical technique, resulting in a favorable outcome.

## 2. Case Presentation

**Figure 1:**
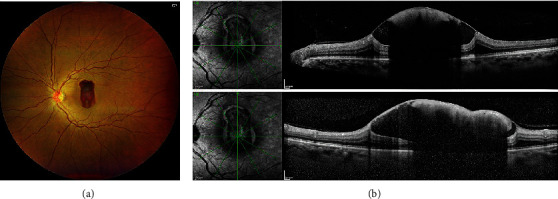
(a) Funduscopic images showing a submacular hemorrhage involving the macula in the left eye at the first visit. (b) Optical coherence tomography images showing subinternal limiting membrane hemorrhage in the left eye.

A 48-year-old female patient was referred to our clinic the same day for further evaluation and treatment of rapid-onset bilateral visual impairment following a breath-holding maneuver during a computed tomography scan. The initial best-corrected decimal visual acuity (BCVA) in the right and left eyes was 1.2 and 0.03, respectively. A slit-lamp examination revealed no abnormality in the anterior segment. Fundus examination revealed the presence of subfoveal sub-ILM hemorrhage in both eyes. The fovea was visible in the right eye, despite the presence of hemorrhage. However, the fovea was completely covered by the sub-ILM hemorrhage in the left eye (Figures 1(a) and 1(b)), making detailed observation challenging. Considering the patient's strong desire for treatment and the bilateral onset, intravitreal injection of recombinant tPA (GRTPA, Tanabe Seiyaku Co., Ltd., Osaka, Japan; 40,000 IU) was performed during the first visit in the left eye; the patient was then instructed to rest in the supine position for the entire day. This was followed by an intravitreal injection of 100% sulfur hexafluoride (SF6) gas (0.4 cc) the next day, with instructions to rest in the prone position to displace the subfoveal hemorrhage in the left eye. The patient was reexamined 1 week after the initial visit. However, the hemorrhage was not displaced, and the BCVA remained unchanged. Thus, on the same day of the follow-up visit (1 week after the initial visit), PPV was performed to displace the hemorrhage after obtaining consent from the patient.

The surgery was performed under sub-Tenon's capsule anesthesia with 2% lidocaine, followed by standard 27 G PPV with a wide-angle noncontact viewing system (Resight®; Carl Zeiss Meditec AG, Jena, Germany) using the Constellation Vision System (Alcon Laboratories, Fort Worth, TX, United States) (Supporting Information). A core vitrectomy was performed, followed by the creation of a posterior vitreous detachment and a subsequent total vitrectomy. The ILM at the temporal-lower position of the fovea (4–5 o'clock direction) was grasped using 27 G MaxGrip forceps (Griesharber®; Alcon Laboratories), and a fissure was created. A balanced salt solution was sprayed onto the ILM, leading to the dissolution of the subfoveal hemorrhage by tPA, and the hemorrhage was drained into the vitreous cavity. FTMH was ruled out using intraoperative optical coherence tomography after thorough drainage of the subfoveal hemorrhage, and the preservation of the ILM above the fovea was confirmed. Subsequently, the vitreous cavity was filled with 20% SF6 gas, and the surgery was concluded. The patient was instructed to maintain a prone position on the day of the surgery and was allowed to assume positions other than the supine position from the next day onward.

**Figure 2:**
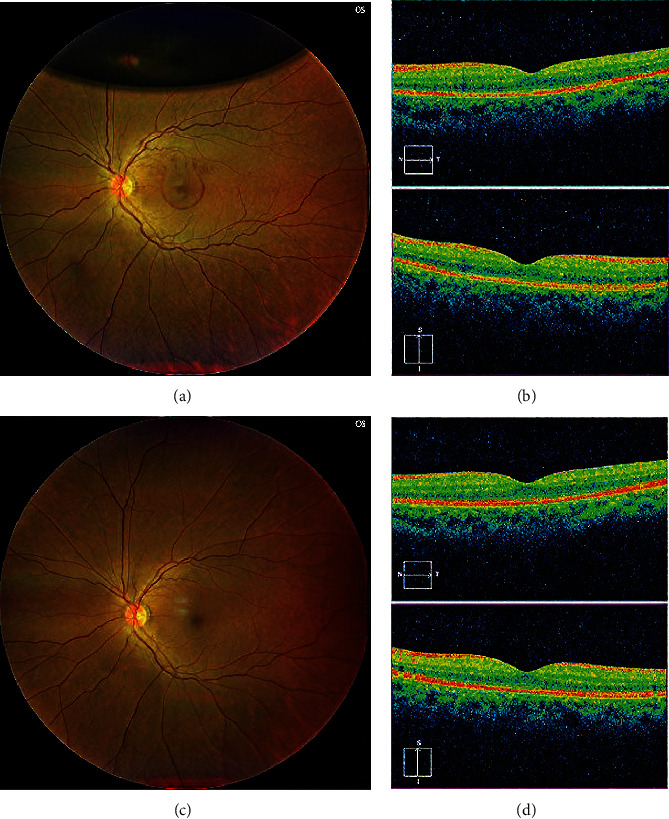
(a) Fundus examination showing absorption of the submacular hemorrhage with no postoperative complications, such as full-thickness macular holes (FTMHs) in the left eye 1 week after the surgery. (b) Optical coherence tomography (OCT) images showing the disappearance of the submacular hemorrhage and the absence of FTMH formation. (c) Recurrence of submacular hemorrhage was not observed 6 months postoperatively. (d) Submacular hemorrhage and FTMH were not observed on the OCT image.

Optical coherence tomography findings confirmed the disappearance of subfoveal hemorrhage and the absence of FTMH formation 1 week postoperatively (Figures 2(a) and 2(b)). The BCVA remained at 1.2 in the right eye and further improved to 1.2 in the left eye at six postoperative months, and no postoperative complications, including FTMH, were observed (Figures 2(c) and 2(d)).

## 3. Discussion

In this case report, we achieved favorable results by performing a surgical technique that removed sub-ILM hemorrhage without peeling the foveal ILM for a patient with SMH secondary to Valsalva retinopathy. We have previously reported this surgical technique for the treatment of sub-ILM hemorrhage secondary to RAM rupture [12]. The current report suggests that this technique may also be effective for removing sub-ILM hemorrhage while preserving the foveal ILM, even in cases of SMH secondary to Valsalva retinopathy.

The advantages of the technique we reported include the following. First, the use of tPA to dissolve the hematoma can loosen its adhesion to the ILM. While tPA is known to be cytotoxic [13], it is necessary to determine the optimal dosage and timing to maximize its benefits and minimize its drawbacks. In this case, the dosage used was considered appropriate, as there have been no reported complications due to tPA in the literature [14]. Regarding timing, if this surgery is planned, it is preferable to operate as soon as possible after tPA administration to minimize its cytotoxic effects, though the best timing is still under debate and requires further investigation. Second, this technique is not complex, is relatively simple, and allows 100% preservation of the foveal ILM.

The etiology of SMH includes various conditions, such as Valsalva retinopathy, and FTMH can occur during any of these diseases [15–18]. The mechanism of FTMH development remains largely unknown; however, generally, the incidence of FTMH secondary to various pathologies is reported to be approximately 1% [19]. However, the incidence of FTMH following SMH secondary to RAM rupture is relatively high (approximately 10%). Moreover, the outcomes of PPV with conventional ILM peeling for this condition are poorer than those for idiopathic FTMH, with a low closure rate of approximately 70% [15]. In this case, the SMH was secondary to Valsalva retinopathy, a relatively rare condition; hence, there are no large-scale studies on the treatment outcomes of FTMH following SMH due to this disease. However, considering the aspects of FTMH following SMH, it is likely to be similar to FTMH following RAM rupture, where the outcomes of PPV with conventional ILM peeling for this condition can be poor. Therefore, developing new surgical techniques for FTMH secondary to SMH is an urgent challenge.

Hence, it is beneficial to preserve the central foveal ILM using this new surgical technique because it enables the closure of FTMH under SMH and potentially prevents the occurrence of postoperative FTMH. Recently, various membrane transplantation techniques, such as the inverted ILM flap technique, autologous ILM transplantation, and ILM repositioning technique, have been reported to be effective for treating refractory FTMHs [20–28]. The mechanism of action of membrane transplantation involves the presence of glial cells, such as Müller cells, within the ILM flap that promotes FTMH closure, and the production of various growth factors by the flap further facilitates glial cell-mediated closure of FTMH [29–31]. Although the methods may differ, it is clear that positioning the ILM on the macula is crucial for promoting the closure of existing FTMH in all techniques. However, membrane transplantation is technically challenging and can sometimes fail in clinical practice, resulting in the nonclosure of FTMH. In this regard, we believe that this surgical technique, which allows for 100% preservation of the central foveal ILM relatively easily, has significant advantages compared to other conventional membrane transplantation techniques, making it a potential alternative to more technically demanding membrane transplantation procedures.

Although intraoperative OCT in this case did not confirm the presence of FTMH, and thus the technique might not have been necessary, it is important to note that FTMH can occur during the natural course of SMH or after various treatments [32–34]. Therefore, preserving the ILM even when FTMH is not confirmed intraoperatively can be useful in preventing postoperative FTMH, making this technique highly advantageous.

In conclusion, we described a case of SMH secondary to Valsalva retinopathy, where subfoveal sub-ILM hemorrhage was removed without foveal ILM peeling. This technique, which enables the preservation of the foveal ILM, may be a viable option for treating SMH.

## Data Availability

The data that support the findings of this study are available from the corresponding author upon reasonable request.

## Supporting Information

Supporting Information Additional supporting information can be found online in the Supporting Information section. Video demonstrating the surgical technique used to preserve the foveal ILM and remove the sub-ILM hemorrhage in this patient.
